# Epigenetic Regulation of Adipokines

**DOI:** 10.3390/ijms18081740

**Published:** 2017-08-10

**Authors:** Tho X. Pham, Ji-Young Lee

**Affiliations:** 1 Department of Nutritional Sciences, University of Connecticut, 216 Advanced Technology Laboratory Building, 1392 Storrs Road, Storrs, CT 06269, USA; tho.pham@uconn.edu; 2 Department of Nutritional Sciences, University of Connecticut, 211C Advanced Technology Laboratory Building, 1392 Storrs Road, Storrs, CT 06269, USA

**Keywords:** adipokines, epigenetics, obesity

## Abstract

Adipose tissue expansion in obesity leads to changes in the expression of adipokines, adipocyte-specific hormones that can regulate whole body energy metabolism. Epigenetic regulation of gene expression is a mechanism by which cells can alter gene expression through the modifications of DNA and histones. Epigenetic mechanisms, such as DNA methylation and histone modifications, are intimately tied to energy metabolism due to their dependence on metabolic intermediates such as S-adenosylmethionine and acetyl-CoA. Altered expression of adipokines in obesity may be due to epigenetic changes. The goal of this review is to highlight current knowledge of epigenetic regulation of adipokines.

## 1. Introduction

Adipose tissue is an important endocrine organ that can regulate whole body energy metabolism by secreting hormones called “adipokines” [1,2,3]. It is known that the level of adipokines in circulation are altered in such conditions as obesity and lipodystrophy [3]. The obesity epidemic is considered largely due to increased consumption of high caloric foods and a sedentary life style that reduces energy expenditure. However, data from animal and human epidemiological studies suggest that interactions between genetic and environmental factors, which are underpinned by epigenetic mechanisms, may play a role in the development of obesity and metabolic disorders [4,5]. Epigenetics refers to the study of changes in gene expression that is heritable but occurs without changes to DNA sequence [6]. Central to epigenetics are modifications to DNA and histones that alter gene expression but also includes microRNA [6]. The subject of this review is to provide comprehensive overview on the epigenetic regulation for the expression of adipokines.

## 2. Epigenetic Mechanisms

### 2.1. DNA Methylation

DNA methylation is perhaps one of the most well-known epigenetic modifications. Methylation at the CpG dinucleotide may increase or decrease expression of methylated genes, depending on the location of the methylation. Methylation near transcriptional start sites of a gene may block initiation of transcription, while methylation in gene bodies does not block initiation, but may even stimulate expression [7,8]. Gene repression mediated by DNA methylation may be attributed to direct inhibition of transcription factor binding or is mediated by methyl-binding proteins that recruit chromatin modifying complexes, such as histone deacetylases (HDACs) [9,10,11].

DNA methyltransferases (DNMTs), including DNMT1, DNMT3a, and DNMT3b, are responsible for the methylation of DNA [12]. It is believed that DNMT1 plays a role in maintaining methylation patterns through replication, while DNMT3a and DNMT3b are responsible for de novo CpG methylation [13]. Of the three types of epigenetic mechanisms, i.e., DNA methylation, histone modification, and microRNAs, the heritability of DNA methylation is best understood. During the semi-conservative process of DNA replication, a parental DNA strand containing 5-methylcytosine serves as the template for the synthesis of a daughter strand. This yields double-stranded DNA containing a parental strand that is still methylated and a daughter strand that is unmethylated, called hemi-methylated DNA. DNMT1 recognizes hemi-methylated DNA and then methylates cytosine residues on the daughter strand to maintain methylation state of DNA through replication [14]. 

DNA methylation was originally thought to be irreversible, but passive and active processes may alter DNA methylation states [15]. Passive DNA demethylation refers to a loss of methylated cytosine through successive rounds of DNA replication in the absence of maintenance of DNA methylation by DNMT1; and active DNA demethylation indicates enzymatic removal or modification of methylated cytosine [15]. Currently, there are no known enzymes that can mediate the direct removal of a methyl group from cytosine. However, 5-methylcytosine can be converted to 5-hydroxymethylcytosine by the ten-eleven translocation (TET) family of enzymes [15]. It is proposed that 5-hydroxymethylcytosine then is recognized by the DNA repair system as a damaged base, which is subsequently replaced by an unmethylated cytosine by the base excision repair system or the nucleotide excision repair system [15,16].

### 2.2. Histone Modifications

In eukaryotic cells, DNA wraps around histone octamers consisting of two units of histone H2A, H2B, H3 and H4, which form the basic unit of chromatin, the nucleosome [17]. Intra and inter-nucleosomal interactions affect chromatin compactness, which ultimately regulates accessibility of DNA to transcription factors [18,19]. Histones undergo post-translational modifications, which affect intra and inter-nucleosomal interactions and, thus, the compactness of chromatin. Histone modifications include acetylation, phosphorylation, methylation, glycosylation, ubiqutination, biotinylation, sumyolation, ADP-ribosylation, palmitoylation, succinylation, and malonlyation [20,21,22,23]. Of these modifications, histone acetylation was first discovered [24] and best characterized, while much less is known about other modifications regarding enzymes that mediate the modification and their physiological significance. The acetylation of lysine on histone tails neutralizes positive charge of lysine residues, which weakens charge-dependent histone–histone interactions and the interaction between histones and the negatively charged backbone of DNA [20]. Therefore, acetylation of histones typically relaxes chromatin, increasing the accessibility of DNA to transcriptional machinery for gene transcription.

The acetylation of histones is mediated by histone acetyltransferases (HATs) while deacetylation is mediated by HDACs. HATs encompass a diverse family of enzymes that are categorized into several families based on sequence homology, structure, and functions [25]. Notable HAT families include the MOZ (monocytic leukemic zinc finger), Ybf2/Sas3, Sas2, and Tip60 (MYST) family, p300/CREB-binding protein (CBP) family, general control non-derepressible 5 (Gcn5)-related N-acetyltransferase (GNAT) family, and the steroid receptor coactivators (SRC) family [26,27]. HDACs are grouped into two families: the zinc-dependent HDACs of the Rpd3/Hda1 family and the NAD+ dependent HDACs of the sirtuin (SIRT) family. HDACs are also categorized into four classes [28]. Class I HDACs include HDAC1, 2, 3, and 8. Class II HDACs include HDAC4-7, 9 and 10. Class III HDACs are SIRT1 through 7, and the only member of class IV is HDAC11 [28,29].

## 3. Role of Energy Metabolism in the Regulation of Epigenetics

Energy metabolism and epigenetic modifications are closely tied together through metabolic intermediates. The rate of histone acetylation is dependent on intracellular levels of acetyl-CoA, which is the acetyl group donor. Acetyl-CoA is compartmentalized in cells and the concentration of acetyl-CoA varies between each compartment [25]. While mitochondrial acetyl-CoA is used for energy production, cytoplasmic and nuclear acetyl-CoA is used for histone acetylation by HATs [30]. Modulation of intracellular acetyl-CoA levels in these cellular compartments affects histone acetylation. Reduction of cytosolic acetyl-CoA via knockdown of ATP-citrate lyase, which is responsible for the synthesis of cytosolic acetyl-CoA, reduced acetylated histones in human HCT116 colon cells [30]. Besides acetyl-CoA levels affecting histone acetylation, histone deacetylation by HDACs is also affected by intermediates of energy metabolism. Acetyl-CoA, malonyl-CoA, and 3-hydroxy-3-methylglutaryl-CoA have been reported to act as allosteric activator of HDACs while free CoA inhibits HDAC activity [31]. Sirtuins, the class III HDACs, require NAD+ as a cofactor for histone deacetylation. NAD+ plays an essential role in energy generation by increasing mitochondrial oxidative phosphorylation and NAD+ levels have been suggested as a limiting factor for optimal mitochondria function [32]. Thus, the availability of NAD+ affects both histone deacetylation and cellular energy metabolism. This further supports the strong link of histone acetylation states to energy metabolism. 

Methylation of DNA and histones is also connected to energy metabolism. Methylation reactions are dependent on S-adenosylmethionine (SAM), a major methyl group donor for cytosines of DNA or lysine and arginine residues of histone tails [33]. As both serine and threonine have been demonstrated to contribute to SAM synthesis, amino acids play an important role in DNA methylation [34,35]. 

## 4. Obesity and DNA Methylation 

Several studies have reported that DNA methylation states are different in the adipose tissue of lean and obese humans. In women who underwent gastric bypass surgery and had a significant weight loss, hypermethylation of CpGs in the adipose tissue was diminished after surgery [36]. Furthermore, differential methylation was found within genes associated with obesity, epigenetic regulation, and embryonic development such as cholesteryl ester transfer protein, forkhead box protein P2, HDAC4, DNMT3b, and Hox genes [36]. Altered methylation of these genes were associated with changes in their mRNA expression [36]. Similarly, weight loss due to a six-month exercise intervention in healthy men altered DNA methylation in subcutaneous adipose tissue and altered gene expression in one-third of the gene regions with altered DNA mutilation [37]. Another study reported differential DNA methylation of gene promoters in the adipose tissue of lean and obese patients [38]. These studies provide evidence that changes in DNA methylation may contribute to altered gene expression in the adipose tissue of obese individuals.

Increased expression of DNMTs in the adipose tissue is associated with changes in DNA methylation in obesity. DNMT1 expression was reported to increase in the adipose tissue of obese mice compared to lean counterparts, and it was also positively correlated with body mass index (BMI) in human adipose tissue [39]. In db/db mice, expression of DNMT3a was significantly increased compared to lean controls [40]. Consistent with increased DNMT1 and DNMT3a expression in the adipose tissue of obese mice, enrichment of DNMT1, DNMT3a, and DNMT3b at the promoter of leptin in the adipose tissue of obese mice was higher than that of lean mice [41]. Interestingly, plasma concentrations of SAM in humans were increased in response to overfeeding and the increase was proportional to fat mass gained [42]. Together, studies have suggested that DNA methylation is differentially regulated in lean and obese adipose tissue. Changes in the expression of DNMTs and changes in tissue SAM levels may alter DNA methylation, which ultimately could affect gene expression in the adipose tissue.

## 5. Epigenetic Regulation of Adipokines

### 5.1. Leptin

Leptin, the first identified adipokine, was discovered serendipitously at the Jackson Laboratories in 1994 [43]. The leptin or “obese” gene codes for a 167-amino acid protein in adipocytes, and it regulates food intake and energy expenditure. Mice harboring a homozygous mutation in the leptin gene are hyperphagic, extremely obese, and diabetic [43,44]. 

Leptin mediates its effect by binding to leptin receptors that are present in the brain and peripheral tissues, including the liver, the heart, the kidneys, the lungs, and the adipose tissue [45]. Through alternative RNA splicing, a single leptin receptor gene gives rise to six leptin receptor isoforms (LEPRa, b, c, d, e, and f) [46]. LEPRb is the only isoform that contains a full length intracellular domain and is thought to be the main leptin receptor that transduces leptin signaling [47,48,49]. LEPRa and LEPRe are known to mediate leptin transport across the blood–brain barrier [50,51,52]. The binding of leptin to its receptor activates several signaling pathways, including phosphatidylinositol 3-kinase (PI3K), Janus kinase-signal transducer and activator of transcription-3 (STAT3), and mitogen-activated protein kinase pathways, among others [53]. In the hypothalamus, binding of leptin to its receptors on pro-opiomelanocortin (POMC) neurons and agouti-related protein (AgRP) neurons modulate appetite. In POMC neurons, leptin signaling stimulates the synthesis of anorexigenic (appetite-suppressing) neuropeptides while leptin inhibits the synthesis of orexigenic (appetite-stimulating) neuropeptides in AgRP neurons [54].

It was originally thought that leptin could be used as a powerful hormone to facilitate weight loss in obese individuals due to its role in suppressing satiety. However, overweight and obese patients already have elevated plasma leptin levels [55,56]. These patients develop leptin resistance, which is defined by the reduced ability of leptin to suppress appetite and weight gain, due to impaired leptin transportation through the blood–brain barrier and dysfunctional leptin receptor signaling [54].

Leptin expression is altered in obesity and is associated with changes in the methylation of its promoter. In the epidydimal fat of DIO (diet-induced obese) mice, methylation in the leptin promoter was significantly reduced compared to low-fat control at eight weeks; but it was significantly increased at 12 and 18 weeks [41]. The data suggest that through DNA methylation, a high fat diet may induce DNA demethylation of the leptin promoter early on to increase leptin expression, possibly to reduce food intake. Consistent with the notion of a feedback mechanism, the epidydimal fat of the DIO mice at 18 weeks showed an enrichment of DNMT1, HDAC1, HDAC2, and HDAC6 in the leptin promoter while acetylated histone H3 and H4 were decreased compared to the control [41]. Although the epigenetic markers suggest a possible decrease in the transcription of leptin, leptin mRNA levels were increased, suggesting other factors may play a role in leptin expression [41]. Recently, it was demonstrated that a prolonged high fat diet induced gradual and fat-specific DNA hypermethylation of leptin and peroxisome proliferator-activated receptor γ (PPARγ) promoters in gonadal, but not subcutaneous fat [57]. Together, these studies demonstrate that a prolonged high fat diet could lead to hypermethylation of the leptin promoter in the adipose tissue.

While DNA methylation has been associated with the expression of leptin, the role of histone deacetylation by HDACs in leptin signaling has been suggested. DIO mice had a significant reduction in hypothalamic expression of HDAC5 compared to lean controls, and injection of leptin to leptin deficient ob/ob mice increased hypothalamic HDAC5 expression [58]. Furthermore, pharmacological and genetic inhibition of HDAC5 increased food intake, whereas hypothalamic overexpression of HDAC5 reduced food intake [58]. It was demonstrated that HDAC5 enhances leptin signaling by deacetylating STAT3 and increasing its nuclear localization and transcriptional activity [58]. Moreover, the activity of HDAC5 and HDAC6 has been implicated in proper adipocyte function; and obese humans and mice exhibit impaired HDAC5 and six activities [59]. Leptin also has anti-inflammatory effects in the adipose tissue, which is mediated through HDAC4, inhibiting nuclear factor κB (NF-κB) for the repression of inflammatory gene expression [60]. Taken together, leptin expression in the adipose tissue may be regulated by DNA methylation, while HDACs are involved in leptin signaling.

### 5.2. Adiponectin

Adiponectin is produced and secreted almost exclusively by adipocytes [61]. Adiponectin is present in the circulation at a very high level ranging between 2 and 30 μg/mL in humans, accounting for ~0.01% of total plasma proteins [62,63,64]. Three adiponectin receptors have been identified: AdipoR1, AdipoR2, and T-cadherin [65,66]. AdipoR1 is ubiquitously expressed, but it is most abundant in skeletal muscle while AdipoR2 is highly expressed in the liver [66]. The binding of adiponectin to its receptor in skeletal muscle and the liver enhances insulin sensitivity by promoting glucose utilization and increasing fatty acid oxidation through the activation of AMP-activated protein kinase and PPARα [67,68]. Decreases in circulating adiponectin levels have been associated with obesity, insulin resistance, and type 2 diabetes [69,70]. 

Downregulation of adiponectin in obesity has been attributed to a decrease in its transcription [69,71]. Recent studies have suggested that epigenetic mechanisms may also play a role in the downregulation of adiponectin in an obese state. In the adipose tissue of DIO mice, adiponectin promoter was hypermethylated and the expression and activity of DNA methyltransferase 1 (DMNT1) in the adipose tissue were increased compared to lean controls, suggesting that hypermethylation of adiponectin promoter may be due to increased DMNT1 [39]. Consistent with this pathological role of DMNT1 in obesity, inhibition of DNA methylation with RG108, a chemical inhibitor of DNA methylation, increased circulating adiponectin levels and improved insulin sensitivity in db/db mice [39]. Tumor necrosis factor α (TNFα) and interleukin-1β (IL-1β) were responsible for the increased DMNT1 expression and activity, suggesting that adipose tissue inflammation may drive the hypermethylation in the adiponectin promoter. In severely obese humans, DNA methylation in the adiponectin promoter was positively correlated with BMI and waist circumference [72]. Furthermore, in the subcutaneous adipose tissue of these individuals, DNA methylation was negatively correlated with adiponectin mRNA levels [72]. Together, these data suggest that DNA methylation plays an essential role in the repression of adiponectin in obesity.

### 5.3. Secreted Frizzled Related Protein 5 (SFRP5)

SFRP5 (Secreted Frizzled Related Protein 5), most abundantly expressed in white adipose tissue of mice, is a soluble modulator of Wnt signaling [73]. SFRP5 is downregulated in the adipose tissue of ob/ob and DIO mice, and in the visceral adipose tissue of obese humans [73]. SFRP5-deficient mice display impaired glucose metabolism and increased fatty liver when fed a high fat diet, which is likely due to increased inflammation in the adipose tissue as SFRP5 suppresses Wnt5a-mediated activation of c-Jun N-terminal kinase 1 (JNK1) [73]. In contrast to the proposed role of SFRP5 as an anti-inflammatory factor in the previous study, it was shown that SFRP5-deficient mice were resistant to diet-induced obesity and had smaller adipocytes in adipose tissue [74]. Adipocytes of SFRP5-deficient mice had increased PPARγ coactivator-1α activity and mitochondria oxidative phosphorylation, which contributed to decreased serum leptin and increased glucose tolerance and insulin sensitivity when mice were on a high fat diet [74]. This study supports a role in which SFRP5 inhibits Wnt signaling and consequently suppresses oxidative metabolism and stimulate adipose growth during obesity. The contrasting findings between the aforementioned two studies may be due to how the SFRP5-deficient mice were generated: The former study replaced the first exon of SFRP5 with a selection cassette, but the latter used mutagenesis to produce a stop codon at glutamine 27 of the *SFRP5* gene.

It was reported that mRNA expression of *SFRP5* in the epididymal fat of C57BL/6J mice prior to being on a high fat diet was highly variable [75]. Initial *SFRP5* gene expression correlated positively with adiposity after eight weeks on a high fat diet; and higher initial expression of *SFRP5* led to higher weight gain on a high fat diet [75]. In addition, in C57BL/6J mice, a high fat diet for four weeks significantly increased the expression of *SFRP5* in the inguinal fat pad [76]. DNA methylation or histone acetylation at the SFRP5 promoter in the inguinal fat pad was marginally altered by the high fat diet, although DNA methylation was significantly increased by age [76]. The data suggest that DNA methylation of the SFRP5 promoter in the adipose tissue is mainly altered during the development, and the initial expression level of SFRP5 is a strong determinant of weight gain once mice are put on a high fat diet, although a high fat diet alone may not alter DNA methylation at the SFRP5 promoter.

### 5.4. Tumor Necrosis Factor α

Early studies demonstrated that adipocytes are capable of secreting several pro-inflammatory cytokines, including IL-1β, IL-6, and TNFα [77,78]. This discovery led to the hypothesis that TNFα produced from adipocytes may relate obesity to insulin resistance as plasma TNFα levels were positively correlated with insulin resistance [79,80]. Binding of TNFα to its receptor on adipocytes can inhibit adipocyte differentiation [81]. Inhibition of adipocyte differentiation by TNFα is mediated by the inhibition of PPARγ activity, which is known as the master regulator of adipocyte differentiation, via the activation of NF-κB [82,83,84]. TNFα also impairs insulin signaling in adipocytes by activating the PI3K-AKT pathway, which phosphorylates hormone-sensitive lipase (HSL) to increase the hydrolysis of triglycerides in adipocytes [85,86]. Insulin signaling induces the dephosphorylation of HSL for inactivation, therefore facilitating fat storage [87]. Thus, TNFα induces dysregulation in adipocytes by facilitating lipolysis while inhibiting adipocyte differentiation, which eventually promotes ectopic lipid storage in other tissues.

The expression of TNFα in the adipose tissue has been tied to several HDACs. It has been demonstrated that TNFα induces the translocation of HDAC3 to the nucleus in preadipocytes to repress PPARγ activity [88]. This suggests that HDAC3 may play an essential role in mediating the effects of TNFα in the adipose tissue. Contrary to the role of HDAC3 in adipocytes, leptin signaling reduces the expression of inflammatory genes, such as TNFα, by increasing nuclear translocation of HDAC4 in adipose tissue macrophages, where it deacetylates histones present on the promoters of inflammatory genes such as TNFα for transcriptional repression [60]. It appears that TNFα expression and signaling are regulated by several HDACs. Therefore, the modulation of HDAC activity with HDAC inhibitors may be able to reduce the adverse effects of TNFα in the adipose tissue.

## 6. Conclusions

Adipose tissue regulates whole body energy metabolism by secreting various adipokines. The expression of several adipokines are under epigenetic regulation, in particular through DNA methylation and histone acetylation. As obesity is associated with dysregulation of the expression of several adipokines, it is possible that epigenetic modifications may play a central role in the regulation of adipokine expression. Table 1 summarizes the association of epigenetic factors and the potential effect on the expression of each adipokine. Unfortunately, our understanding of how the expression of many adipokines is regulated by epigenetic modes is very limited. On the few adipokines where epigenetic regulation data do exist, only the most common epigenetic modifications, such as DNA methylation and histone acetylation, are investigated. More studies are needed to understand the types of epigenetic modifications that are involved in the regulation of adipokine expression in obesity. This will help identify therapeutic targets for the treatment of obesity-related diseases that are largely influenced by adipokines.

## Figures and Tables

**Table 1:** Adipokines’ expression in obesity and association with epigenetic factors.

Adipokine	Epigenetic Factor	In Obesity	mRNA Expression in Obesity
Leptin	DNA methylation	Increase at promoter	Increase
	DNA methyltransferase 1	Increase at promoter	Increase
	Histone deacetylase 1	Increase at promoter	Increase
	Histone deacetylase 2	Increase at promoter	Increase
	Histone deacetylase 6	Increase at promoter	Increase
Adiponectin	DNA methylation	Increase at promoter	Decrease
Secreted frizzled related protein 5	DNA methylation	Unchanged	Increase
	Histone acetylation	Increase at promoter	Increase
Tumor necrosis factor α	Histone deacetylase 3	Not available	Increase
	Histone deacetylase 4	Not available	Increase
